# Germanium-embedded bioactive fabric reduces bacterial bioburden and modulates fibroblast and macrophage behavior *in vitro*

**DOI:** 10.3389/fbioe.2026.1823301

**Published:** 2026-06-23

**Authors:** Nicholas J. Tucker, Lyndah Chow, Peter Linde, Gina Myers, Steven Dow, Nicholas A. Alfonso, Lynn M. Pezzanite

**Affiliations:** 1 Department of Orthopedics, Anschutz Medical Campus, University of Colorado, Aurora, CO, United States; 2 Department of Clinical Sciences, Orthopedic Research Center, Translational Medicine Institute, College of Veterinary Medicine and Biomedical Sciences, Colorado State University, Fort Collins, CO, United States; 3 Immunotherapy Research Laboratory, Department of Clinical Sciences, Translational Medicine Institute, College of Veterinary Medicine and Biomedical Sciences, Colorado State University, Fort Collins, CO, United States; 4 Incrediwear Holdings, Inc., Chico, CA, United States

**Keywords:** bioactive fabric, germanium, germanium embedded fabric, infection, surgical site infection

## Abstract

**Introduction:**

Surgical site infections (SSIs) occur in 2%–4% of all surgical cases. Wearable bioactive fabrics (BAFs) containing semiconductor germanium are reported to promote microcirculation, enhance immunity, and increase antibacterial activity, with the potential to improve outcomes in SSIs. While BAFs are proposed to exert their effects through the generation of negative ions and the emission of far-infrared radiation, their mechanism of action has not been fully explored. The objective of this study was to evaluate the impact of BAFs on bacterial bioburden, fibroblast migration and proliferation, and inflammation modulation in the context of SSIs.

**Methods:**

Utilizing an *in vitro* study design, BAF was compared to control fabric (CF; non-bioactive, without inclusion of semiconductors) and untreated (UT) groups using indirect assays in which the BAF was not in direct contact with cells or bacteria but was instead separated by an air gap. These assays examined the effects of BAF proximity on bacterial killing (*Staphylococcus aureus* ATCC 25923 and *Escherichia coli* ATCC 25922), fibroblast cell confluence, wound density, macrophage cytokine production, and macrophage differential gene expression.

**Results:**

BAF exposure reduced *S. aureus* bacterial bioburden compared to CF and UT (*p* < 0.0001) and reduced *E. coli* bioburden compared to UT (*p* < 0.0001). Wound density, indicating fibroblast migration was greater in the BAF group compared to CF and UT at 24 and 48 h (*p* = 0.03 and *p* < 0.01, respectively). Fibroblast proliferation was reduced in the BAF group compared to UT at 18 and 36 h (*p* = 0.04 and *p* = 0.01, respectively). Cytokines IL-10, IL-13, TNF-β, and PDGF were elevated in culture supernatants of macrophages treated with BAF compared with CF and UT (*p* < 0.05). IL-1β and IL-17A were elevated with BAF vs. CF treatment macrophages (*p* < 0.05), whereas IL-6 and IP-10 were elevated in BAF-treated macrophages compared with UT-treated macrophages (*p* < 0.05). BAFs also altered macrophage gene expression, including genes associated with interferon, innate immunity, and toll-like receptor pathways, compared with CF.

**Discussion:**

Exposure to BAFs exhibited direct and indirect bactericidal effects, enhanced fibroblast migration, reduced fibroblast proliferation, increased immunomodulatory cytokine secretion, and altered macrophage gene expression in ways that may positively impact wound healing. BAFs warrant further *in vivo* investigation to reduce SSIs and enhance healing.

## Introduction

Surgical site infections (SSIs), defined as those developing within 30 days of surgery or 1 year after orthopedic implant placement, are associated with longer lengths of hospital stay (LOS), higher readmission rates, greater total medical costs, and higher mortality rates (Shambhu et al., 2024; Rezaei et al., 2025; Smyth and Emmerson, 2000; Seidelman et al., 2023; Stolz et al., 2025). SSIs occur in 2%–4% of all surgical cases in the United States with up to 30% incidence in specific procedures (Shambhu et al., 2024; Rezaei et al., 2025; Smyth and Emmerson, 2000). Currently employed strategies to prevent or reduce SSIs include the use of clippers for preoperative hair removal, skin decolonization with anti-*Staphylococcal* antiseptics or intranasal agents, chlorhexidine gluconate and alcohol-based skin preparations, maintenance of normothermia through active warming to a core body temperature >36 °C, perioperative glycemic control (<150 mg/dL), negative pressure wound therapy, and preoperative systemic antimicrobial prophylaxis (Seidelman et al., 2023). Commonly implicated bacterial isolates include *Staphylococcus aureus*, *Staphylococcus epidermidis*, *Streptococcus* species, *Pseudomonas aeruginosa*, *Escherichia coli*, and *Enterococcus faecalis*; however, SSIs are increasingly polymicrobial and complicated by multidrug-resistant organisms (Rezaei et al., 2025; Worku et al., 2023; Giacometti et al., 2000).

Postoperative wearable textiles, including germanium-embedded bioactive fabrics (BAFs), have been introduced to modulate inflammation and potentially reduce infection (Chen et al., 2022). The biological effects of germanium in biological systems have been proposed to result from far-infrared (FIR) emission, which promotes vasodilation, improves circulation, mitigates inflammation, and demonstrates concentration-dependent antibacterial activity (Chen et al., 2022; Luo et al., 2023; Chung and Lee, 2014; Brattain and Briggs, 1949; Juho et al., 2021). Germanium has a unique atomic nuclear structure that allows the generation of negative ions and the emission of FIR electromagnetic waves, which can be derived from its optical constants (Chen et al., 2022). As a semiconductor, germanium is activated by body heat (temperatures >32 °C), releasing negative ions that may reduce inflammation and oxidative stress by neutralizing free radicals (Chen et al., 2022; Luo et al., 2023; Chung and Lee, 2014; Brattain and Briggs, 1949; Juho et al., 2021; Dalven, 1966; Duranti et al., 2024). The BAF application has further been reported to result in increased blood and lymphatic flow with FIR waves (λ = 3–100 μm subdivision of the electromagnetic spectrum), which may be associated with more rapid wound healing (Chen et al., 2022; Duranti et al., 2024; Vatansever and Hamblin, 2012; Toyokawa et al., 2003; Lin and Li, 2015; Qin et al., 2024). For these reasons, the integration of germanium into BAFs has been suggested to provide potential benefits in musculoskeletal conditions (Duranti et al., 2024; Justice and Jacob, 2024; Lee et al., 2018; Marino et al., 2019). For example, BAFs have been investigated for improving pain and function in the non-operative management of early knee osteoarthritis and enhancing early functional recovery following select surgical procedures (total knee arthroplasty) and professional sports injuries (predominantly lower extremity soft tissue injuries) (Duranti et al., 2024; Justice and Jacob, 2024; Lee et al., 2018; Marino et al., 2019). The BAF application has been associated with a histological increase in collagen regeneration and fibroblast infiltration expressing TGF-β (Vatansever and Hamblin, 2012; Toyokawa et al., 2003; Lin and Li, 2015; Qin et al., 2024). However, studies to date have not fully investigated the cellular and molecular effects of germanium or its incorporation into functional textiles to determine whether the observed treatment effects occur through modulation of immune cell lineages that inhibit progressive inflammation or through alternative mechanisms.

Therefore, the overall aim of this study was to further delineate the mechanism(s) of action of BAFs in the context of infections and tissue healing in postoperative SSIs using an *in vitro* culture system to evaluate its impact on bacterial bioburden, fibroblast migration and proliferation, and inflammation modulation (Chen et al., 2022; Duranti et al., 2024; Vatansever and Hamblin, 2012; Toyokawa et al., 2003; Lin and Li, 2015; Qin et al., 2024). The objective was to further the understanding of the antibacterial and wound-modulating characteristics of germanium-embedded BAF as a potential adjunctive modality for reducing SSIs.

## Methods

### Study overview

**FIGURE 1:**
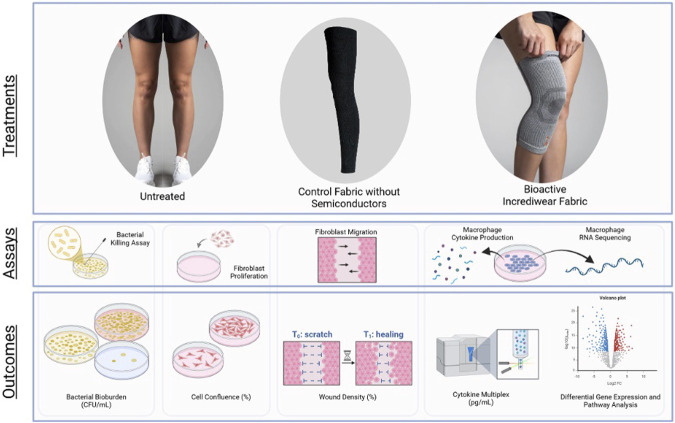
Study overview. The effect of germanium-embedded bioactive fabric technology was assessed in reducing infections and modulating inflammation in wound healing using a series of *in vitro* assays. Bioactive fabric was compared to non-germanium embedded control fabric without semiconductors or no treatment for their effect on direct bacterial killing of Gram-positive and Gram-negative isolates, fibroblast proliferation and migration, macrophage cytokine secretion, and differential gene expression.

The effect of germanium embedded BAF technology was assessed to reduce infection and modulate inflammation in wound healing using a series of *in vitro* assays. BAF was compared to non-germanium-embedded control fabric (CF; without semiconductors) or untreated (UT) groups for its effects on direct bacterial killing of Gram-positive and Gram-negative isolates, fibroblast proliferation and migration, macrophage phagocytosis, cytokine secretion, and differential gene expression. The study overview and product design are shown in Figure 1.

### Bacterial culture

The human *S. aureus* (MRSA) strain ATCC 25923 and *E. coli* strain ATCC 25922 were used in bacterial killing assays (Colorado State University Biological Project Approval Number 25-024B). Bacteria were expanded in Luria-Bertani broth and frozen at −80 °C in 20% glycerol until further use. Overnight bacterial cultures were grown in antibiotic-free complete growth medium (DMEM with 1,000 mg/L glucose, 10% or 1% FBS, and 1 M HEPES) before use in the assays. On the day of the experiment, bacterial sub-cultures were grown to log phase in complete medium (OD600 of 0.6, corresponding to 7.5 log_10_ CFU/mL) and then used immediately.

### *S. aureus* and *E. coli* planktonic bacterial killing assays

To assess the ability of germanium-embedded BAFs to directly kill bacteria, antibiotic-free media (200 μL per well in a 96-well plate) were inoculated with 50 μL of actively dividing log-phase *S. aureus* or *E. coli* (OD600 of 0.6, corresponding to 7.5 Log10 CFU/mL bacteria per well). Treatment groups included BAF, CF, or UT. Plates were incubated at 37 °C for 18 h. Negative control wells containing antibiotic-free growth medium without bacterial inoculation were also included. Following incubation with bacteria, the medium was transferred to 1.5 mL tubes, vortexed to evenly distribute bacteria, diluted 10-fold, plated on Luria-Bertani (LB) agar plates (100 μL per quadrant), and incubated at 37 °C for 18 h. Each treatment was reported as the average of n = 3 technical replicates, and experiments were repeated twice to report a final average of n = 6 technical replicates. Colony-forming units (CFUs) were counted manually.

### Fibroblast migration assays

Normal human dermal fibroblasts (HDFs, Thermo Fisher Scientific, #C00405C) were expanded *in vitro* using media and supplements recommended by the manufacturer. Low-passage fibroblasts were plated on Incucyte® Imagelock 96-well Plates (Sartorius, Göttingen, Germany) and incubated overnight for adherence. A uniform scratch wound was created in each well using the Incucyte® Woundmaker 96-Tool; plates were then sandwiched with germanium-embedded BAFs on the top and bottom of the wells without cell contact and placed in a 37 °C, 5% CO_2_ incubator. This ‘air-gap’ model was used to most closely replicate the clinical topical application of BAFs for alleviating symptoms of inflammation without direct application to open wounds. Images were obtained 24 and 48 h post-wound induction and the Incucyte® Cell Migration Scratch Wound Analysis module was used to calculate wound area and density to determine migration speed.

### Fibroblast proliferation assays

Fibroblasts (HDFs) were plated at a low concentration of 5,000 cells per well in a standard 24-well cell culture plate. Culture plates were then sandwiched with germanium-embedded BAFs on the top and bottom of the well without cell contact and placed at 37 °C in a 5% CO_2_ incubator. Fibroblast proliferation was measured by area using the Incucyte® Proliferation Assay every 24 h for up to 96 h.

### Macrophage transcriptome and cytokine assessment

Human THP-1 cells, an immortalized cell line from human acute monocytic leukemia (ATCC, American Type Culture Collection), were used for macrophage assays in triplicate (Chanput et al., 2014). To assess cytokine secretion and differential gene expression of THP-1 macrophages following treatment, cells were plated at 100,000 cells per well in culture media (Dulbecco’s modified Eagle medium (DMEM) supplemented with 10% fetal bovine serum (FBS)) containing IL-1β and TNF-α at 20 ng/mL. Plates were then sandwiched with BAFs on the top and bottom of wells without cell contact (i.e., air-gap model) and placed at 37 °C in a 5% CO_2_ incubator for 18 h (stimulation and treatment time with BAF). At that time, cells and supernatants were separated via centrifugation (500 g for 10 min), and cell culture supernatants were aliquoted in 200 µL volumes and frozen at −80 °C for subsequent evaluation of cytokine concentrations using a multiplex assay. Cells were washed with phosphate-buffered saline (PBS) and collected in RNA lysis buffer (350 μL/sample) and frozen at −80 °C until RNA isolation was performed.

### Macrophage cytokine quantification

A fluorescent bead-based multiplex assay (Milliplex MAP Human Cytokine/Chemokine Magnetic Beads Multiplex Assay, Millipore Sigma, Burlington, MA, 01803) was used to quantify the concentrations of 10 analytes relevant to wound healing [TNF-α, TNF-β, VEGFA, PDGF-AB/BB, IL-1B, IL-10, IL-6, IL-17A, IP-10, and IL-13] in supernatants according to the manufacturer’s instructions.

### RNA extraction for transcriptomic analyses

RNA was extracted from frozen samples using the RNeasy kit (QIAGEN Germantown, MD) according to the manufacturer’s instructions and sent to Novogene Corporation Inc. (Sacramento, CA) for RNA sequencing. RNA quality was determined using a bioanalyzer (Agilent Technologies, Santa Clara, CA), and all samples had an RNA integrity number (RIN) greater than 9.0. mRNA was enriched using oligo (dT) beads, followed by cDNA library generation using the TruSeq RNA Library Prep Kit (Illumina, San Diego, CA). Sequencing was performed on an Illumina NovaSeq 6000 instrument using 150 bp paired-end reads.

### Data analysis

To assess bacterial killing, colony-forming units (CFUs) were measured during the log phase of bacterial growth and reported as log CFU as this allows a linear mapping of optical density to bacterial counts. Graphical analyses and graph generation were performed using Prism software v8.4.1 (GraphPad Software Inc., La Jolla, California). For all analyses, statistical significance was assessed as *p* ≤ 0.05.

Cytokine data were assessed for normality using Shapiro–Wilk tests and visual assessment of diagnostic plots. The effect of activated supernatant treatment on cytokine production was evaluated using one-way ANOVA followed by *post hoc* Tukey’s adjustment for multiple comparisons (for normally distributed data) or the Kruskal–Wallis test (for non-normally data).

For RNA sequencing data analysis, demultiplexed fastq reads from Novogene Inc. were aligned and analyzed using the Alpine high-performance computing resource at the University of Colorado Boulder (University of Colorado Boulder Research Computing, 2023). Fastq files were quality-filtered, and adapters were removed using fastp v0.23.2, then aligned to the human genome assembly GRCh38.p14, and annotated with Ensembl version 114 using STAR v 2.7.10b. Differential expression analysis was computed using DESeq (Love et al., 2014). Volcano plots were generated using ggplot, and heatmaps were generated using pheatmap in Rstudio (2024.04.0 + 735 “Chocolate Cosmos” Release) (Wickham, 2016; Kolde, 2026). For pathway analysis, edgeR differential expression of log_2_ fold change results were used to perform GSEA Preranked analysis (Hallmarks, Reactome, WikiPathways, PID, and KEGG) (Love et al., 2014; Wickham, 2016; Kolde, 2026; Martin, 2011; Anders et al., 2015; Subramanian et al., 2005).

## Results

### Germanium-embedded BAFs induced direct bacterial killing of Gram-positive and Gram-negative bacterial isolates

**FIGURE 2:**
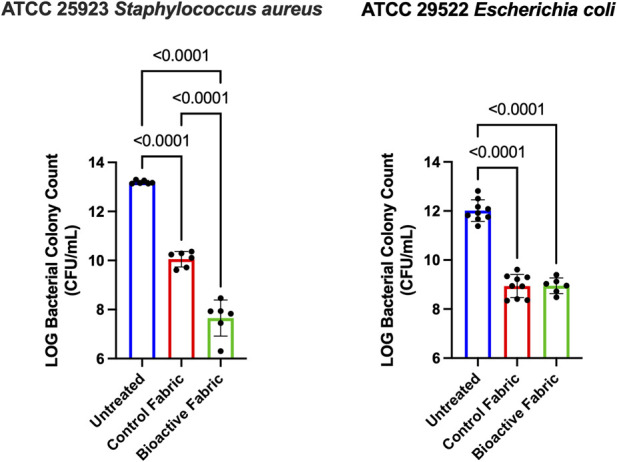
Bactericidal activity against planktonic *S.* and *E. coli* bacteria. The human *Staphylococcus aureus* (MRSA) strain ATCC 25923 and *Escherichia coli* strain ATCC 25922 were used in bacterial killing assays. Overnight bacterial cultures were grown in antibiotic free media, and on the day of the experiment, bacterial sub-cultures were grown to log-phase in the MSC medium (OD600 of 0.6, corresponding to 7.5 log_10_ CFU/mL) and then used immediately. Treatment groups included bioactive fabric, control fabric, or no treatment. Plates were incubated at 37 °C for 18 h. Negative control wells containing antibiotic-free growth medium without bacterial inoculation were also included. Following incubation with bacteria, the medium was transferred to 1.5 mL tubes, vortexed to evenly distribute bacteria, diluted 10-fold, plated on Luria–Bertani (LB) agar plates (100 μL per quadrant), and incubated at 37 °C for 18 h. Each treatment was reported as the average of n = 3 technical replicates, and experiments were repeated three times to report a final average of n = 9 technical replicates. CFUs were counted manually. Significance was assessed at *p* < 0.05 (indicated by *).

Co-culture with BAFs induced direct bacterial killing of both Gram-positive and Gram-negative isolates. When bacterial killing was assessed against *S. aureus*, BAFs reduced bacterial colony counts relative to CF and UT (*p* < 0.0001). When bacterial killing was assessed against *E. coli*, BAFs reduced bacterial colony counts relative to UT (p < 0.0001) (Figure 2).

### Germanium-embedded BAFs enhanced fibroblast migration ability while suppressing proliferation

**FIGURE 3:**
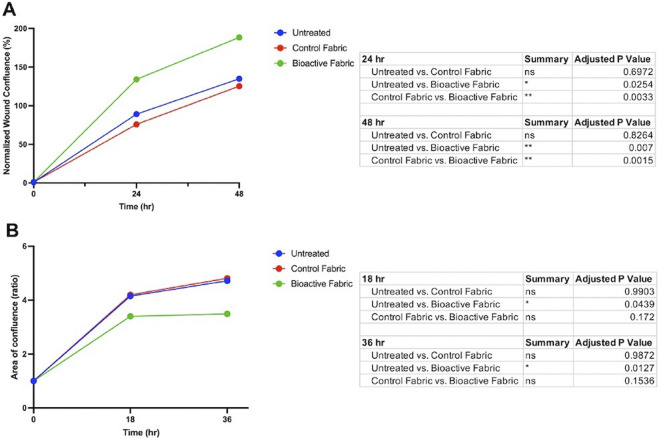
Fibroblast migration and proliferation. **(A)** Percent wound density as measured by the Incucyte® scratch wound assay. The x-axis represents time in culture, and the y-axis shows the percentage of scratch space occupied by fibroblast, normalized to the starting point in each well. Each point shows the mean of five technical replicates. Statistical summary of significant time points (day 1 and day 2) in the table on the right. **(B)** Percent confluence of fibroblast incubated with bioactive or control fabrics (without direct contact) versus no treatment over 36 h. The x-axis shows time, and the y-axis shows the area of confluence on the cell culture plate, normalized to starting confluence. Measured by Incucyte. Treatment groups are shown in color labels. The table shows significance summary.

To simulate wearable products in the clinical setting, fabric was placed on the top and bottom of cell culture plates and incubated at 37 °C in 5% CO_2_ to replicate body heat and activate the emission of infrared radiation and negative ions underlying the bioactive effects observed in germanium embedded textiles. BAFs increased the migration capability of fibroblasts in culture relevant to wound healing compared to UT and CF at 24 (*p* = 0.03 and *p* = 0.003, respectively) and 48 h (*p* = 0.007 and *p* = 0.002, respectively) (Figure 3A). BAF resulted in suppression of fibroblast proliferation compared to UT after 18 h (*p* = 0.04) and 36 h (*p* = 0.01) (Figure 3B).

### Germanium-embedded BAFs enhanced monocytic cytokine secretion

**FIGURE 4:**
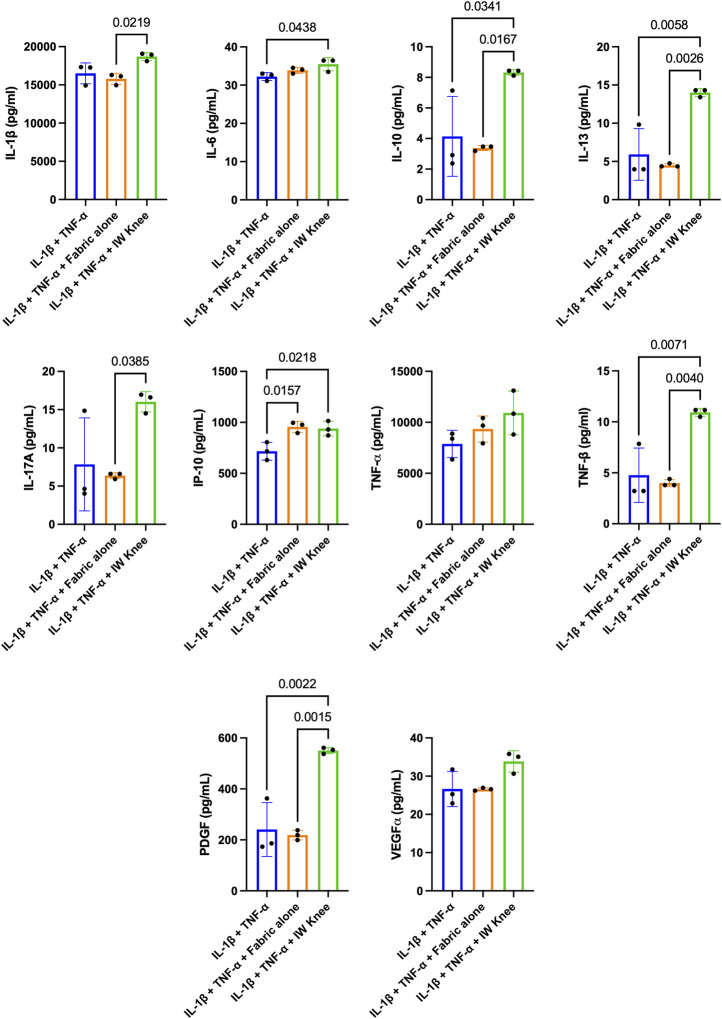
Effect of germanium-embedded bioactive fabrics on macrophage cytokine secretion. Fluorescent bead-based multiplex assay (Milliplex MAP Human Cytokine/Chemokine Magnetic Beads Multiplex Assay, Millipore Sigma, Burlington, MA, 01803) was used to quantify the concentrations of 10 analytes [TNF-α, TNF-β, VEGFA, PDGF-AB/BB, IL-1B, IL-10, IL-6, IL-17A, IP-10, and IL-13] in macrophage supernatants following bioactive or control fabric exposure. Results are reported in pg/mL; statistical comparison is presented without non-stimulated macrophage controls; * significance was assessed at *p* < 0.05.

The impact of BAFs on the secretion of 10 cytokines relevant to wound healing from stimulated innate immune effector cells (THP1 monocytic cell line readout system) was evaluated. Results were analyzed both with (Supplementary Document 1) and without (Figure 4) the inclusion of non-stimulated control cells. Findings indicate that BAFs stimulate macrophage activation with the upregulation of cytokines relevant to innate immune cell induction and promotion of healing (IL-10, IL-13, IP-10, PDGF, and TNF-β) and those related to inflammation (IL-17, IP-10, and IL-6). Specifically, cytokines IL-10, IL-13, TNF-β, and PDGF were elevated in culture supernatants of cells treated with BAF compared to CF and UT (*p* < 0.05); IL-1β and IL-17A were elevated with BAF compared to CF treatment (*p* < 0.05); and IL-6 and IP-10 were elevated with BAF compared to UT treatment (*p* < 0.05).

### Germanium-embedded BAFs impacted monocytic differential gene expression

**FIGURE 5:**
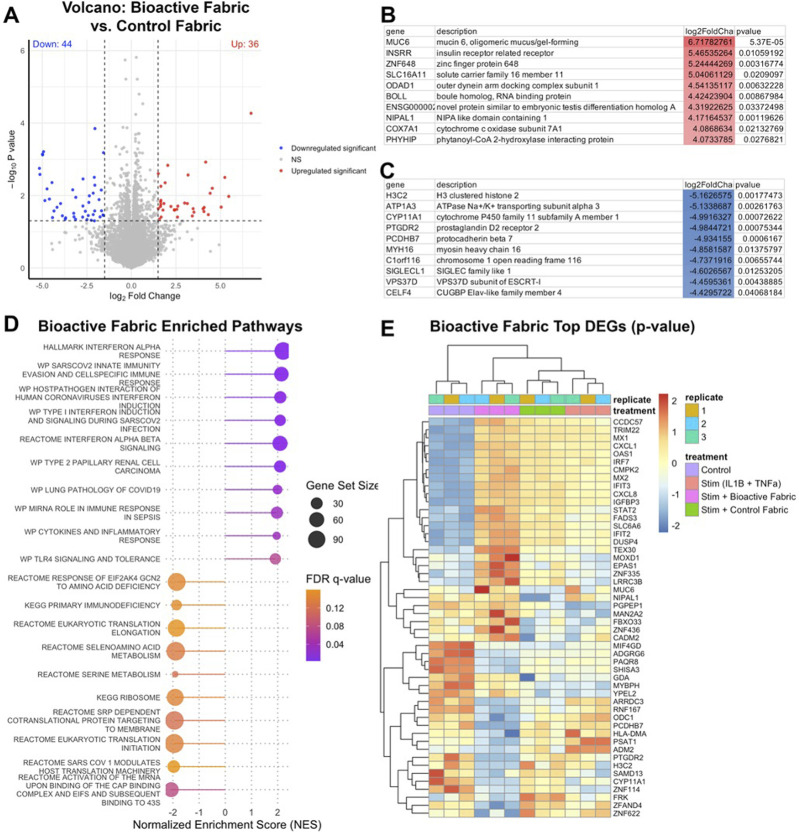
Effect of bioactive fabric on macrophage gene expression and pathway analyses. **(A)** Volcano plot of DESeq differential analysis results. Significantly upregulated genes are shown in red, and significantly downregulated genes are shown in blue. **(B)** Gene list of the top 10 significantly upregulated genes (by FC) in bioactive fabric exposed THP-1 macrophage. **(C)** Gene list of the top 10 significantly downregulated genes by FC. **(D)** Top 20 enriched GSEA pathways in bioactive fabric exposed THP-1 macrophage with FDR (false discovery rate) adjusted *p*-value ≤ 0.25. **(E)** Heatmap with unsupervised hierarchical clustering of the top 50 DEGs (differentially expressed genes) by *p*-value in bioactive vs. control fabric. Treatment groups are labeled in color.

To simulate the wound environment, THP-1 macrophages were treated with inflammatory cytokines TNF-α and IFN-β. The transcriptome (mRNA) expression of THP-1 macrophages was compared after exposure to BAF or CF. Overall, the differences with BAF were mild compared to the differences between unstimulated THP-1 macrophages and the THP-1 macrophages with the addition of inflammatory stimuli IL-1β and TNF-α (Figure 5).

When comparing the gene expression of the BAF-exposed THP-1 macrophages to the control fabric, there were a total of 80 significantly differentially expressed genes (Figure 5A); the top genes by highest and lowest (most downregulated) fold change are listed (Figures 5B,C). Out of the top 50 most significant genes ranked by *p*-value, macrophage function genes such as CXCL8, CXCL1, and HLA and interferon-regulated genes such as IFIT and OAS were impacted by BAF, resulting in an immune-activated phenotype compared to CF treatment (Figure 5E). To summarize the gene expression differences, inflammatory pathways such as interferon alpha, immune response, and toll-like receptors (TLRs) were upregulated, while some pathways related to protein synthesis are downregulated (Figure 5D). Patterns in interferon pathway genes indicate that BAFs induced overall higher expression of interferon (types I and II) genes, which could have beneficial effects on short-term wound healing as early interferon expression is associated with promotion of wound debris clearance, increased antimicrobial activity, and enhanced monocyte and neutrophil recruitment.

## Discussion

This study evaluated the impact of germanium-embedded BAFs in the context of treating or preventing SSIs using an *in vitro* study design to assess its effects on bacterial bioburden, fibroblast migration and proliferation, and inflammation modulation. In the United States alone, SSIs are estimated to add between $3.3 and $10 billion to the annual cost of care (Shambhu et al., 2024; Rezaei et al., 2025). Database studies utilizing commercial and Medicare Advantage/Supplement claims have shown that SSIs are associated with increased hospitalization length of stay by 1.7–6.3 days, higher 30-day readmissions (odds ratio 2.8–25.1), increased mortality (hazard ratio 1.6 among orthopedic spine patients), and increased total cost of medical care by average 1-year differences from $40,606 to $68,101 per patient (Shambhu et al., 2024). These findings demonstrate that BAFs reduced Gram-positive and Gram-negative bacterial bioburden through a direct bactericidal effect, increased fibroblast migration while suppressing proliferation, and enhanced secretion of immunomodulatory cytokines, most notably IL-10, IL-13, TNF-β, and PDGF related to wound healing. BAFs further altered differential gene expression, upregulating pathways related to interferon alpha, immune response, and TLRs, resulting in an immune-activated phenotype, which may be key to enhancing innate immune effector cells in the early perioperative healing phase to help reduce infection. These findings suggest that BAFs may play a role in promoting wound healing in the postoperative surgical setting to reduce the risk of SSIs.

First, the findings reported here support and expand upon previous literature reporting the inherent antimicrobial properties of germanium-embedded fabrics (Chen et al., 2022). Chen et al. (2022) demonstrated excellent antibacterial activity of germanium fibers against *S. aureus*, with bacterial colony reduction rates higher than 90% at all concentrations investigated and as high as 99.6%, was improved with higher concentrations of germanium particles (up to 6% by weight). Following that work, the theorized mechanism of action included the emission of negative ions from activated germanium that were able to readily adsorb to Gram-positive bacteria and neutralize their biological activity, leading to cell death, which was not observed with Gram-negative *E. coli* (Chen et al., 2022). Chung and Lee (2014) built upon this work by evaluating the far-infrared (FIR) properties of poly (vinyl alcohol) (PVA) membranes filled with germanium and silicon dioxide nanoparticles, demonstrating that the membranes exhibited 99.9% bacterial reduction against *S. aureus* and *E. coli* but diminished efficacy (34.8%) against *Klebsiella pneumoniae*. Ji Y. et al. (2023) provided further evidence for antibacterial activity against both *S. aureus* and *E. coli*, as well as biocompatibility following incorporation of germanium in functionalized composite materials in combination with silicocarnotite. This study adds to the growing body of literature investigating the impact of functional bioactive fabrics containing germanium as the primary active agent on common bacterial isolates relevant to SSIs.

The findings of this work further reinforced the concept of a potentially enhanced effect against Gram-positive vs. Gram-negative isolates, indicating that differences in the cell wall and outer lipid membrane of resident or target bacteria in SSIs may influence the impact of BAF treatment. However, as *S. aureus*, including methicillin-resistant and coagulase-negative strains, remain one of the most common bacterial isolates implicated in surgical SSIs, BAF exposure may be uniquely positioned as an adjunctive postoperative treatment to potentially help reduce SSI without introducing the risk of antimicrobial resistance. Current options for primary dressings over surgical wounds include, but are not limited to, standard gauze dressings, antimicrobial dressings (silver, iodine, and polyhexamethylene biguanide), films, foams, hydrogels, hydrocolloids, and negative pressure wound therapy. Systematic reviews and meta-analyses have reported largely inconclusive data, with no single dressing option demonstrating superiority (Dumville et al., 2016; Jiang et al., 2020). Although there may be some benefit to vitamin E-silicone and mupirocin-containing dressings in preventing SSI, this finding has not been consistently replicated (Jiang et al., 2020). Further investigation of BAFs is indicated to assess their efficacy as a primary dressing in comparison to or as a supplement to traditional surgical dressings.

In addition, the impact of BAFs on fibroblast migration indicates their potential not only to directly reduce local bacterial bioburden but also to promote tissue healing in surgical incisions. Findings of this study indicated that BAF treatment enhanced fibroblast migration compared to CF or UT while suppressing proliferation, which may have important implications for encouraging cells to move into the wound area to repair tissue and promote more rapid wound contraction while minimizing granulation tissue or scar formation (fibrosis). Matsumoto et al. (2016) observed a similar wound healing response with the use of an organic germanium compound (Ge-132) for dermal injuries in a rat model, reporting reduced wound area at 7 and 14 days, decreased edema and increased fibroblast migration at 7 days, and increased macrophage activity at 14 days (Matsumoto et al., 2016). These findings were associated with increased expression of TGF-β1 and α-SMA mRNA at 24 h (Matsumoto et al., 2016). Prior literature provides evidence that other fabrics, particularly electrospun nanofiber scaffolds, can achieve a similar balance by promoting fibroblast migration while suppressing proliferation, a behavior that is crucial for wound healing applications (Woodley et al., 2023; Berry et al., 2024). However, further *in vivo* investigation of BAFs is warranted to determine the biological consequences of this dual role and optimal timing of application as some degree of fibroblast proliferation is also necessary for tissue repair.

One proposed mechanism for this observation is that germanium may protect human dermal fibroblasts from cellular death caused by oxidative stress in a dose-dependent manner (Takeda et al., 2019). Rather than suppressing cell death by scavenging reactive oxygen species such as general antioxidants, germanium has been theorized (based on gene expression analysis) to regulate genes related to cellular death, including suppressed expression of the nuclear receptor subfamily 4 group A member 2 (NR4A2) gene related to cell death and suppressed expression of IL-6 and chemokine ligand 2 (CXCL2) genes related to the inflammatory response (Takeda et al., 2019). An additional potential explanation is that these findings are an effect of far-infrared emission from germanium activation as FIR has been found to accelerate wound healing and be associated with increased infiltration of fibroblasts expressing TGF-β1 (Vatansever and Hamblin, 2012; Toyokawa et al., 2003). Further evaluation of the impact of BAFs on these pathways would enhance understanding of the direct effects of germanium-embedded BAFs on fibroblasts at varying stages of wound healing to tailor the application of BAF dressings to the stage of tissue healing following insult, trauma, or surgical incision.

Finally, these findings revealed key insights into the immunological impact of BAFs on direct immunomodulatory cytokine secretion and differential gene expression of macrophage-like cells that are key to the early response to tissue healing. Specifically, cytokines IL-10, IL-13, TNF-β, and PDGF were present at higher concentrations in secreted supernatants of THP-1 macrophages treated with BAF than in the supernatants of those treated with CF or UT; cytokines IL-1β and IL-17A were present at higher concentrations than in the CF group, while cytokines IL-6 and IP-10 were present at higher concentrations than in the UT group. The concurrent increase in pro-inflammatory and anti-inflammatory cytokines reflects the complex inflammatory milieu in wound healing. IL-6 plays a multifaceted role, initially promoting inflammation and increasing immune response at the wound. Then, in subsequent phases, IL-6 switches to promote tissue repair through macrophage polarization and angiogenesis. Imbalances in IL-6 levels at different stages of healing can lead to chronic wounds or impaired healing (Johnson et al., 2020). Similarly, IL-17 drives early inflammation to reduce bacterial burden in the wound and clear debris, but dysregulation of the IL-17 axis can result in chronic inflammation and disease (Gaffen, 2009). Upregulation of these inflammatory cytokines may be beneficial initially for treating infection in SSIs; however, prolonged elevation may lead to chronic inflammation and delayed wound healing. These findings potentially indicate that early application of BAFs during the tissue healing process may be advantageous, whereas prolonged treatment may delay tissue healing, warranting further clinical evaluation.

In contrast, when predominant, IL-10 and IL-13 shift healing from a pro-inflammatory state to one of tissue repair, both promoting macrophage polarization toward a reparative M2 phenotype and extracellular matrix remodeling (Cioce et al., 2024; Steen et al., 2020; King et al., 2014; Sun et al., 2020; Singampalli et al., 2020; Wong et al., 2025; Allen, 2023). IL-10 specifically promotes scarless healing through the induction of hyaluronan (ground substance) reducing fibrosis in the wound bed (Steen et al., 2020; King et al., 2014; Sun et al., 2020), while IL-13 stimulates fibroblasts to promote extracellular matrix (ECM) formation, primarily collagen synthesis by fibroblasts critical for tissue repair (Wong et al., 2025; Allen, 2023). In recent years, IL-10 has been the focus of potential antifibrotic therapies, with the implication that the development of novel therapeutics, such as BAF, that capitalize on targets within the IL-10 signaling pathway could have substantial implications for patients either suffering from fibrosis or to minimize loss of skin structural integrity and function prior to pathologic fibrotic tissue accumulation and scarring (Steen et al., 2020). In fetal tissue, IL-10 has been shown to play an essential role in the ability of the fetus to heal regeneratively with scarless tissue, which has been hypothesized to be the result of pleiotropic effects through regulation of the inflammatory response related to ECM, fibroblast cellular function, and endothelial progenitor cells (King et al., 2014). Further studies investigating the timing, dose, and route of IL-10-focused treatments such as BAFs are warranted to promote their potential as anti-scarring therapeutics (Steen et al., 2020; Sun et al., 2020). IL-13 has also been shown to regulate extracellular matrix modification and remodeling for effective tissue repair (Allen, 2023).

Additionally, platelet-derived growth factor (PDGF) has been described to promote tissue repair through the recruitment of macrophages and neutrophils, stimulating the migration of fibroblasts and smooth muscle cells to the wound bed and fibroblast synthesis of collagen, glycosaminoglycans, and other matrix components for tissue scaffolding to generally promote angiogenesis, epithelialization, and wound contraction (Qin et al., 2025; Zarei and Soleimaninejad, 2018; Pierce et al., 1991; Jian et al., 2022; Lynch et al., 1987). Controlled PDGF delivery has been shown to augment wound breaking strength, accelerate time to closure, enhance neovascularization, and promote wound closure in preclinical models (Zarei and Soleimaninejad, 2018; Pierce et al., 1991).

Finally, the upregulation of interferon-alpha and innate immune pathways in BAF-treated THP-1 macrophages indicated potentially critical implications to promote bacterial reduction through immune cell (natural killer, macrophage, and dendritic cell) activation, chemokine (CXCL10) recruitment of dendritic cells to the wound bed to kill exposed bacteria, and induction of interferon-stimulated genes (ISGs), leading to bacteriolysis (Qiu et al., 2025; Ji L. et al., 2023; Xia et al., 2025; Di Domizio et al., 2020). However, upregulation of IFN-α plays a complex role in inflammation regulation, initially promoting inflammation to clear pathogens while having negative effects when sustained, indicating that further investigation of timing and duration of BAF application in the temporal course of wound healing is indicated (Qiu et al., 2025; Ji L. et al., 2023; Xia et al., 2025; Di Domizio et al., 2020). In summary, exposure to BAFs increased immunomodulatory cytokine secretion and altered macrophage gene expression in ways that may positively impact wound healing.

While these findings may indicate a potential benefit of germanium-embedded BAFs in bacterial reduction and tissue healing relevant to the early stages of surgical site healing, several caveats to study design deserve mention. Limitations of this study include the *in vitro* design, lack of mechanistic validation, and indirect (‘air-gap’) exposure model, warranting further investigation and confirmation in preclinical models and clinical trials to assess safety and impact. Specific to the antimicrobial effect, the impact of BAFs on various bacterial species implicated in SSIs was not exhaustively performed, and further *in vivo* assessment of their effect on Gram-negative isolates is warranted to determine whether extended duration or route of application may enhance or compromise effects. As discussed above, the complex effect of BAFs on the immunological cascade relevant to incisional healing indicates a need for further evaluation of timing and duration of application of wearable textiles to optimize impact while minimizing potential deleterious effects (including promotion or prolongation of the inflammatory phase).

Further justification for and emphasis on the clinical relevance of the air-gap model warrants discussion. The germanium-embedded fabrics evaluated in this study are registered as Class I medical devices with the FDA for use in alleviating symptoms of pain and edema and improving functional outcomes. The fabric has not been cleared or approved for use in treating disease or for direct application to open wounds. In the clinical setting, the products do not come into direct contact with tissue other than the epidermal layer of skin. This guided the experimental approach to expose the culture environment indirectly to the fabric. In terms of the mechanism of activation of the technology, the air-gap model is further relevant in that the fabric is thought to be activated by heat energy and emits infrared radiation and negative air ions through electron excitation and mobilization. The experimental approach of exposing the fabric to temperatures of 37 °C in the incubator and exposing the cellular environment and media to the fabric simultaneously may reinforce the concept that the effects of the semiconductor technology within the fabric are through the emission of infrared radiation and negative air ions, rather than interactions with the physical components of the fabric itself, with the media or cells. Limitations of the air-gap model approach are acknowledged as head-to-head comparisons with direct contact or varied exposure times were not evaluated, and the proposed mechanism of infrared (IR) and negative air ion (NAI) emission was not directly measured or visualized in this study. The proposed mechanism of action is thought to be that the fabric is activated by heat energy and the emission of infrared radiation and negative air ions through electron excitation and mobilization based on prior discussion of the role of germanium in disease and its biological effects (Luo et al., 2023). It is acknowledged that direct experimental validation with the fabrics evaluated here would strengthen the mechanistic understanding of the specific fabrics.

These findings suggest that transient early application in an open wound environment or the postoperative period may provide benefit to reducing bacterial bioburden and promote wound healing by recruitment of innate immune cells and fibroblast migration, but further evaluation is needed to define parameters of clinical application in the context of specific cases and in light of other commercially available wound dressing options.

## Conclusion

In summary, this study reported a multi-dimensional analysis (microbial, cellular, and transcriptomic) of the effects of germanium-embedded BAFs to exert both direct and indirect bactericidal effects and modulate resident tissue cell lineages key to promoting wound healing. BAF treatment enhanced fibroblast migration, increased immunomodulatory cytokine secretion, and altered macrophage gene expression *in vitro*. These findings suggest that BAFs may play a role in promoting tissue healing in the postoperative surgical setting, with the potential to reduce the risk of SSIs. Further investigation of the appropriate timing during wound healing and case selection for application *in vivo* is warranted.

## Data Availability

The datasets presented can be accessed in the online repository NCBI GEO public genomic data repository with accession number GSE334700.

## Supplementary material

SUPPLEMENTARY DOCUMENT 1 Cytokine analyses including control/non-stimulated macrophages. Fluorescent bead-based multiplex assay (Milliplex MAP Human Cytokine/Chemokine Magnetic Beads Multiplex Assay, Millipore Sigma, Burlington, MA, 01803) was used to quantify the concentrations of 10 analytes [TNF-α, TNF-β, VEGFA, PDGF-AB/BB, IL-1B, IL-10, IL-6, IL-17A, IP-10, and IL-13] in macrophage supernatants following bandage or calf sleeve exposure. Results reported in pg/mL; statistical comparison presented with non-stimulated macrophage controls; * significance was assessed at *p* < 0.05.
