# Supplementary figures and images for: Germanium-embedded bioactive fabric reduces bacterial bioburden and modulates fibroblast and macrophage behavior in vitro
Source: Front Bioeng Biotechnol. 2026 Jun 23;14:1823301. doi: 10.3389/fbioe.2026.1823301 (PMC13338619; doi:10.3389/fbioe.2026.1823301)

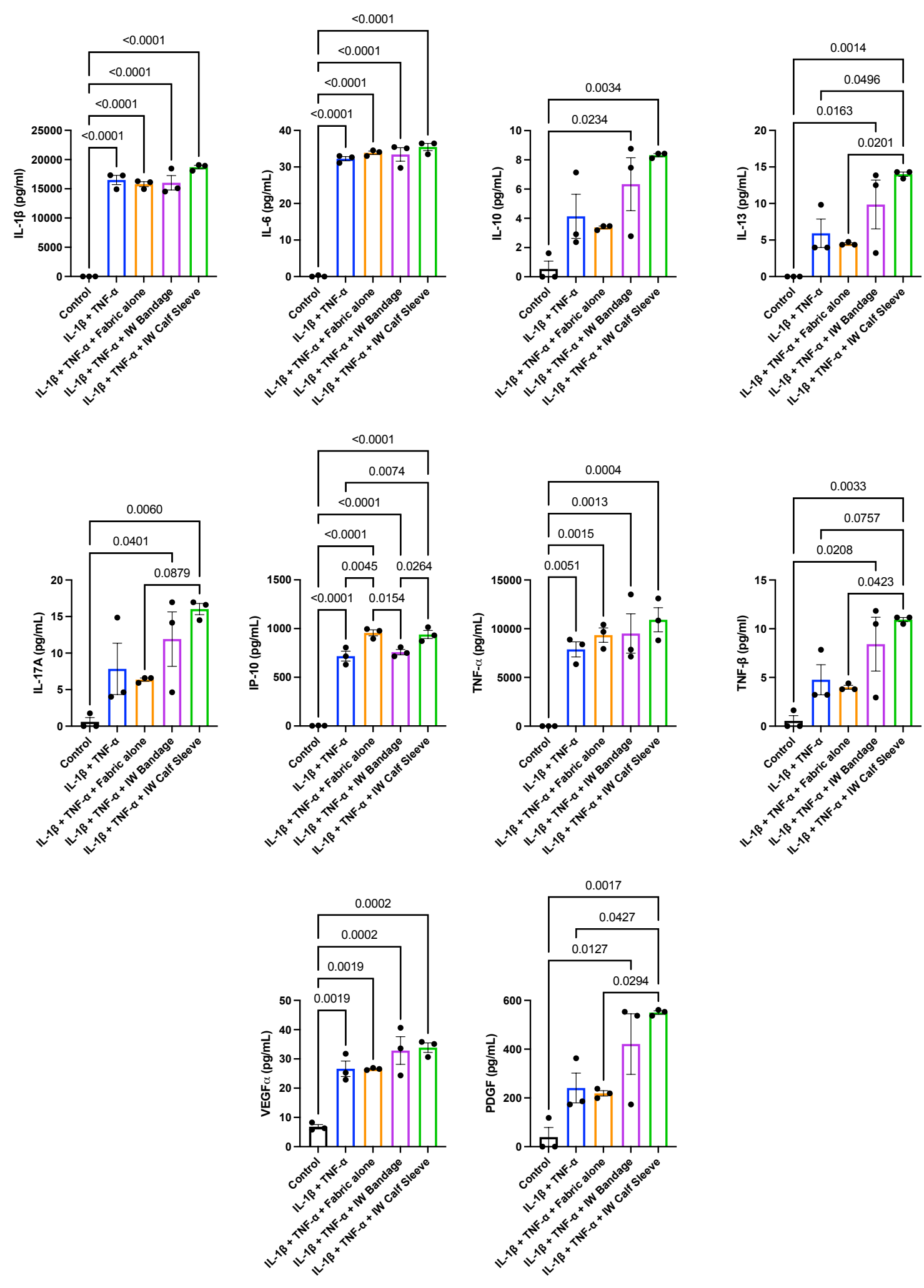

Supplement: Supplementary file 1 [file Image1.pdf]
